# Isolation of extracellular vesicles: Determining the correct approach (Review)

**DOI:** 10.3892/ijmm.2015.2194

**Published:** 2015-04-22

**Authors:** RAFAL SZATANEK, JAREK BARAN, MACIEJ SIEDLAR, MONIKA BAJ-KRZYWORZEKA

**Affiliations:** Department of Clinical Immunology and Transplantology, Jagiellonian University Medical College, 30-663 Krakow, Poland

**Keywords:** extracellular vesicles, exosomes, microvesicles, differential centrifugation

## Abstract

The discovery of extracellular vesicles (EVs) has revised the interpretation of intercellular communication. It is now well established that EVs play a significant role in coagulation, inflammation, cancer and stem cell renewal and expansion. Their release presents an intriguing, transporting/trafficking network of biologically active molecules, which are able to reach and modulate the function/behavior of the target cells in a variety of ways. Moreover, the presence of EVs in various body fluids points to their potential for use as biomarkers and prognostic indicators in the surveillance/monitoring of a variety of diseases. Although vast knowledge on the subject of EVs has accumulated over the years, there are still fundamental issues associated with the correct approach for their isolation. This review comprises the knowledge on EV isolation techniques that are currently available. The aim of this review was to make both experienced researchers and newcomers to the field aware that different types of EVs require unique isolation approaches. The realization of this ‘uniqueness’ is the first step in the right direction for the complete assessment of EVs.

## 1. Introduction

Intercellular communication in a multicellular organism is essential for its proper functionality. Traditionally, soluble factors released by different types of cells were considered to serve such a role in a localized environment (1). With the emergence and discovery of small membrane sacs, later termed as extracellular vesicles (EVs), that are released by various cell types, the concept of ‘cell-to-cell talk’ has been re-evaluated (2,3). It is now widely accepted that EVs have a substantial contribution to intercellular communication by shuttling bioactive molecules (transmembrane receptors, mRNAs, miRNAs and signaling molecules) that are able to modulate the extracellular environment (4–9). The term EV implies to all shed membrane vesicles, which can be further classified on the basis of their size, origin and their cargo (10). In general, however, EVs have been divided into two groups, exosomes and microvesicles (5,10,11). Exosomes are formed through invagination into endosomes to form multivesicular bodies (MVBs) and are thought to be somewhat unique in their protein and lipid composition (10). Due to their endosomal origin, exosomes contain membrane transport and fusion proteins (GTPases, Annexins and flotillin), tetraspannins (CD9, CD63, CD81 and CD82), heat shock proteins (Hsc70 and Hsp90), proteins involved in multivesicular body biogenesis (Alix and TSG101), as well as lipid-related proteins and phospholipases (10,12,13), although other markers have also been associated with them (14). On the other hand, microvesicles are particles shed from the plasma membrane following stimulation and are often a hallmark of cell apoptosis (15–17). Upon cell activation, as a result of stimuli, such as shear stress or cytokine/endotoxin release, the cytosolic calcium concentration increases, leading to the activation of a number of enzymes (calpains, gelsolins, scramblases and kinases) (16). This, in turn, leads to the inhibition of enzymes, such as translocases and phosphatases, resulting in cytoskeletal reorganization, the loss of membrane asymmetry and membrane blebbing, which causes microvesicle formation and release (16,18). Microvesicles have a rich phospholipid bilayer consisting of phosphatidyl serine on the outer leaflet and their membrane proteins reflect those of the cell they originate from (15,17,18).

## 2. Concept of EV isolation

The concept of EV isolation begins with the nature of the sample itself. First of all, one has to realize that different samples present unique obstacles when it comes to EV isolation, which is greatly related to the origin of the EV. Thus, with respect to the source, EVs can be divided into two groups: i) those isolated from cell culture media, and ii) those isolated from body fluids (plasma, urine, spinal fluid, saliva, etc.).

## 3. Cell culture medium

**Figure 1:**
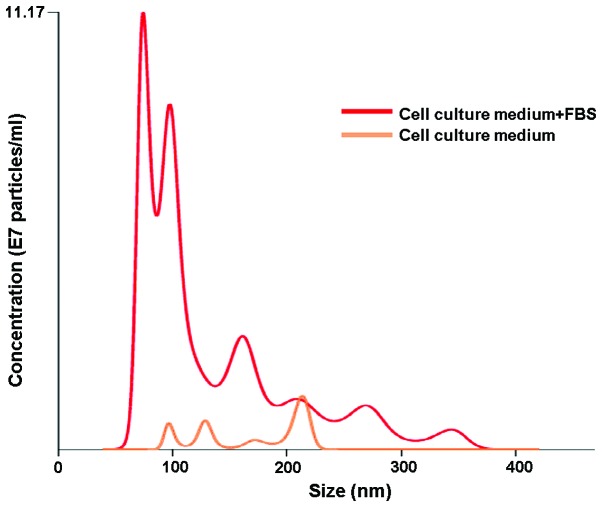
Size distribution of particles obtained by nanoparticle tracking analysis (NTA) from cell culture medium sample supplemented with fetal bovine serum (FBS) and from freshly opened cell culture medium.

When isolating EVs from conditioned cell culture media one has to consider the presence of an additional, ‘artificial’ EV source, namely fetal bovine serum (FBS) or any other supplement for that matter, which is routinely added to cell cultures (19,20). Our results obtained by nanoparticle tracking analysis (NTA) showed freshly prepared cell culture medium supplemented with FBS to have a substantial particle population (mostly <100 nm in size) already present before the actual use (Fig. 1). This observation alone clearly demonstrates that the downstream EV isolation from conditioned cell culture media carries along the risk of obtaining, apart from proper EVs, also those from FBS, which obviously, obscures the final results. This observation is in accordance with observations of other groups, which have also shown FBS to contain vesicles that may be later isolated along the actual EV (19–21). To counter that problem, many groups apply filters to remove EVs from the media/FBS or use a long ultracentrifugation step. In an elegant study, Lötvall *et al* showed that the removal of FBS-originated EVs is critical for further downstream experiments, since these vesicles are capable of inducing effects similar to those of EVs isolated from the actual cell line culture media (21). Thus, it has been proposed that a 16 h-ultracentrifugation step at 100,000 × g or greater for FBS is absolutely obligatory for a complete FBS vesicle depletion, since shorter centrifugation steps are insufficient (19,21). An alternative may be the use of an exosome-free FBS, which is already commercially available, but is still rather expensive. Some also postulate the use of bovine serum albumin (BSA) instead of the standard FBS (20).

Another major issue involving EV isolation from conditioned cell culture media regards the culture medium itself. Our NTA results revealed that even a newly opened cell culture medium contains a trace of particles that resemble EVs size-wise (Fig. 1). The presence of these background particles itself puts tremendous strain on the integrity of the final EV isolation results. Similar results were obtained by Jeppesen *et al*, who compared two different cell culture media and showed that one type had more particle background than the other (19). Moreover, the storage temperature also seems to have an effect on the occurrence of the particles in a cell culture medium. A cell culture medium stored at room temperature showed more background particles than the one stored at 4°C as observed by NTA, which was even more apparent with time passage (own data). It has been suggested that in such cases, ultracentrifugation for an extended period of time (16 h or longer) is necessary for the removal of particle background from the cell culture media (19).

There seems to be a strong demand for establishing a uniform protocol for EV isolation, particularly for EVs which are being isolated from cell culture media; however, this may not be so simple. Studies have shown that different cell lines generate unique EVs, suggesting that they should be considered individually with respect to the isolation approach. In their study, Jeppesen *et al* showed that the optimal isolation conditions for EVs obtained from embryonic kidney HEK293 and bladder carcinoma FL3 cells differed from each other (19). Their study indicated that the optimal vesicle-to-protein yield for HEK293 cells was obtained at 67,000 × g, while that for FL3 cells was at 100,000 × g. Furthermore, it was demonstrated that specific g-force/k-factor usage during differential centrifugation greatly influences the purity and yield of exosomes (19).

## 4. Body fluids

EV isolation from body fluids seems to be even more complex. Although the problem of the FBS vesicle presence obviously does not apply in this case, there are other factors (lipoproteins, DNA, RNA, protein aggregates and microbes) that need to be addressed for proper isolation (22–26). The majority of the available data concerns the overall EV population isolated from blood samples; however, there are attempts to ‘select out’ from that EV pool a certain type of EV depending on its origin (i.e., platelet, epithelial, leukocyte and tumor). Again, a careful approach needs to be undertaken for the isolation of the ‘wanted’ EV fraction(s). For example, if the isolation of leukocyte-origin EV is desired, the sample needs to be depleted of platelets. For that to be accomplished, platelets need to be centrifuged at a certain speed and under appropriate conditions (discussed in further detail below) to avoid platelet activation and, consequently, the generation of EVs of platelet origin (27,28). It has to be kept in mind that a blood sample (or any other body fluid sample for that matter) is a source of many types of EVs. Realizing the complexity of such samples puts another perspective on the EV fraction isolation approaches.

## 5. Storage and isolation conditions

There are no strictly defined conditions for storing/isolating EVs. One exception, however, are platelet-derived EVs, where firm guidelines for their proper acquisition have been laid down by the Vascular Biology group of the Scientific and Standardization Committee of the International Society on Thrombosis and Hemostasis over a decade ago (27–29). Although some basic framework has been established for other EVs, many groups have still independently developed their own storage/isolation protocols that are suitable to their individual laboratory settings (30). There is a general understanding that freshly acquired and processed samples guarantee the best EV yield; however, that is, for the most part, not possible (30,31).

Body fluid samples (blood, serum, plasma, urine and tumor ascites) are collected/stored in a number of ways. For example, there are studies demonstrating that blood samples collected on different anticoagulants, when processed further downstream, exhibit different EV yields (27,28). It has been shown that platelet and endothelial EV counts were substantially lower in blood samples collected in citrate or EDTA than in the ones collected in protease inhibitors, either hirudin and soybean trypsin inhibitor or heparin (27–29,32). Some groups follow strict protocols and process their blood samples within 1 h after collection (33). Others believe that a fasting period (of up to 12 h) prior to the sample collection is necessary for proper EV acquisition (27,28,34). On the other hand, others have indicated that storing blood samples, or any other body fluid samples for that matter, at 4°C for up to 5 days does not affect the final EV yield (20). Observations have also been made that the EV diameter significantly decreases with the duration of the storage period (e.g., within 2 days) and with the increasing temperature during storage (from 4–37°C) (35). Freezing and thawing cycles are also critical. Most groups are in agreement that multiple freezing-thawing cycles of a sample affect EV characteristics/concentration, although some suggest that repeating these cycles for up to 10 times has no influence on the size and composition of EVs to any significant degree (29–32,35). Moreover, the thawing conditions alone seem to play a crucial role in EV recovery. It has been observed that EV samples thawed on ice showed a lower EV recovery compared to the ones thawed at room temperature or 37°C (36). To resolve this issue, aliquots of once obtained EV stocks are stored at −70 to −80°C until use for up to a year, which then undergo only one freezing/thawing cycle (20,34). Zhou *et al* reported that the storage of EVs isolated from urine at −20°C resulted in a major loss of EVs, while storage at −80°C had no effect on the EV recovery (37).

In our opinion, the general rule for storage and isolation conditions should be ‘the sooner, the better’. The starting sample should be handled rapidly after collection, avoiding extensive waiting periods between further processing stages (i.e., centrifugation steps). In blood/plasma samples, all the necessary precautions (i.e., processing temperature, upright sample position for transport, no agitation) should be undertaken to avoid platelet activation, and thus potential platelet-derived EV generation. Moreover, once isolated, EV aliquots should be prepared, and they should undergo only one freezing/thawing cycle at an appropriate temperature to ensure optimal EV recovery.

## 6. Isolation

There are basically three major methodologies used for EV purification/isolation that serve as the backbone for potential method variations: i) differential centrifugation/ultracentrifugation with/without a sucrose gradient/cushion; ii) adsorption to magnetic/non-magnetic microbeads; and iii) size exclusion chromatography.

### Differential centrifugation/ultracentrifugation

**Figure 2:**
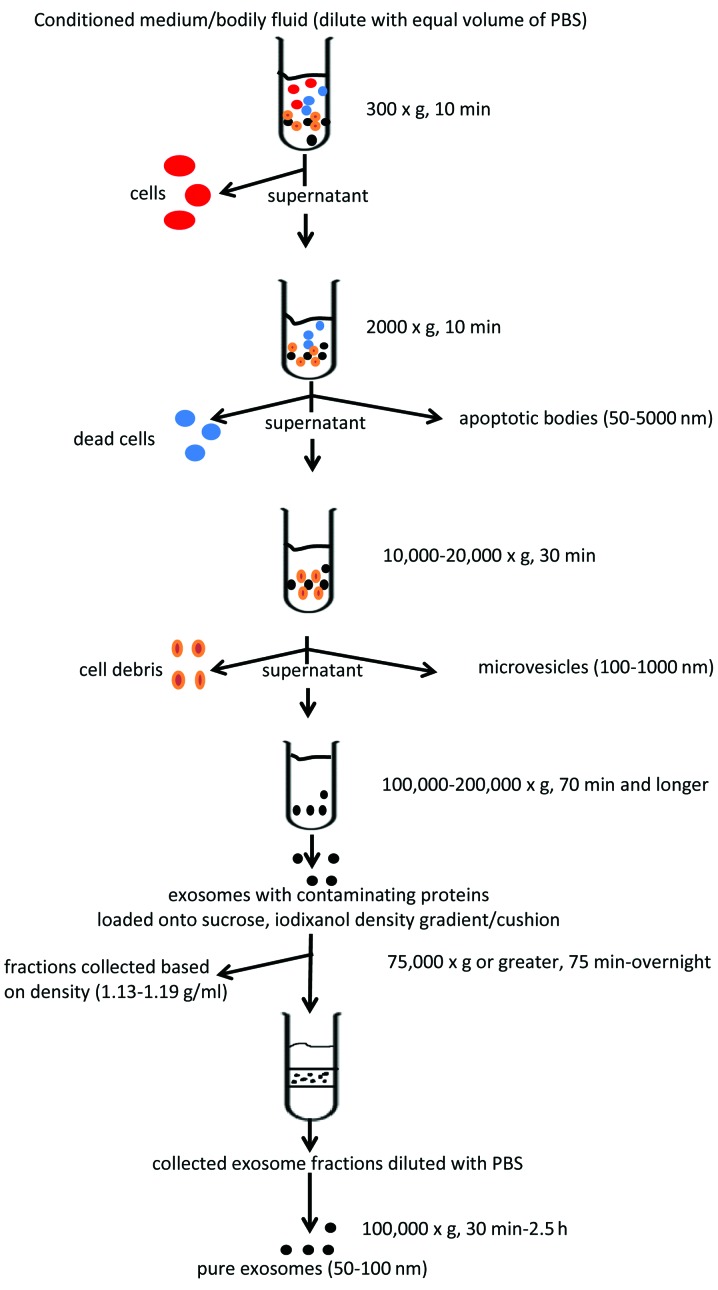
Differential centrifugation scheme, including a sucrose, iodixanol gradient/cushion step.

This is probably the most commonly used method for EV isolation/purification (20,30,38). As with other methods, there are variations depending on the laboratory setting; however, for the most part, the protocols follow the scheme that was put forward in the study by Raposo *et al*, who purified exosomes from the conditioned culture media of transformed human B cell lines (38). The protocol involves a number of sequential centrifugation steps at different centrifugal forces (g) whose purpose is to remove unwanted components from the actual exosomes. The first three steps of the protocol are designed to remove intact cells, dead cells or cell debris using three different centrifugal forces, that is 300 × g for 10 min, 2,000 × g for 10 min and 10,000 × g for 30 min, respectively. After each centrifugation, the supernatant is transferred into a new test tube while the generated pellets are being discarded. After the 10,000 × g spin, the supernatant is then subjected to a final ultracentrifugation at 100,000 × g for 70 min. The outcome of this step is an exosome pellet that can be used for further studies. It should be also noted that all the centrifugation steps are being carried out at 4°C. The basic scheme for EV isolation is presented in Fig. 2.

Although the protocol presented in the study by Raposo *et al* (38) served as the backbone for others, it focused only on the purification of exosomes, a portion of the EV population with a size of <100 nm, and their isolation from conditioned cell culture media. With the discovery of EVs being present in all types of body fluids, there is an increasing need to adopt the protocol for appropriate samples with the incorporation of a step(s) enabling the isolation of EVs with a larger size (>100 nm). The EVs that can be obtained from a body fluid sample are divided into four distinct EV populations: exosomes, microvesicles, apoptotic bodies and microsomes (19). Exosomes (40–100 nm), the most extensively studied EV population, are usually isolated by centrifugation at 100,000–200,000 × g (20,39), whereas microvesicles (100–1,000 nm) are isolated by centrifugation at 10,000–20,000 × g (30,40). Apoptotic bodies (50–5,000 nm) are obtained at a g-force of approximately 2,000 × g (19), whereas microsomes are 80–120 nm in size and their isolation/identification needs to be confirmed by additional means (19,41,42). For the isolation of EVs from plasma, lymph fluid, urine, bronchiolar lavage fluid and tumor ascites, Théry *et al* proposed diluting the samples with an equal volume of PBS before further processing due to the viscosity of the respective fluids (20). Moreover, due to the complexity of the viscous fluid samples the time and centrifugation speeds have been increased/adapted to obtain the appropriate EV populations. The major difference compared to the protocol presented in the study by Raposo *et al* (38) was made at the beginning of the modified protocol. Here, after the initial centrifugation step at 300 × g, another additional centrifugation at 2,000–3,000 × g for up to 30 min is performed, followed by the 12,000–15,000 × g step for up to 1 h. The purpose of the first step is to deplete the sample of cells/cellular debris. The second step enables the acquiring of apoptotic bodies, whereas the third one eliminates platelets/platelet EVs (plasma samples) and/or isolates microvesicles. Some groups at this point check whether platelets/platelet EV have been removed from the centrifuged samples by anti-CD41, -CD61 monoclonal antibody staining and flow cytometry analysis (40). Only platelet-free plasma (PFP) and platelet EV-free plasma samples undergo further processing. It should be noted that, although some groups recommend centrifugation at 4°C at all times, that should not be the case when dealing with plasma samples. The processing of these samples at this temperature leads to platelet activation, which in turn causes the generation of platelet EVs that may become difficult to remove downstream.

A variation of the EV isolation method by centrifugation that has been adopted by some groups includes an additional sucrose gradient/cushion step (43–45). Some argue that during EV isolation by centrifugation, aggregates of large proteins and/or proteins that were non-specifically associated with EVs are also being sedimented (20,45). A sucrose gradient (20–60%)/cushion (30% sucrose) step incorporated into the centrifugation protocol supposedly eliminates this contamination, and the resulting EV population seems to be of a greater purity. The diluted (with PBS) EV suspension obtained by the protocol discussed above is gently loaded onto the Tris/sucrose/D_2_O solution containing tube and ultracentrifuged for a period of time (75 min up to overnight at 75,000 × g or greater) (20,44–47). Following centrifugation, the EV fractions are collected based on their density, that ranges between 1.13–1.19 g/ml, diluted with PBS and centrifuged again for 30 min–2.5 h, at 100,000 × g (20,44–47). Although the incorporation of the density gradient step into the EV isolation protocol has been designed to purify and isolate specific EV fractions, it has been recently reported that certain high-density lipoproteins (HDLs) can also be isolated using this method (48). An alternative to these problems may be the OptiPrep velocity gradient. In this method, 5–40% iodixanol gradient is used instead of sucrose, showing, reportedly, an improved separation of EVs from viral particles and small apoptotic bodies (47). Moreover, unlike sucrose, iodixanol is capable of forming iso-osmotic solutions at all densities, thus better preserving the size of the vesicles in the gradient (46).

### Immunoaffinity isolation

Another method used for EV isolation involves microbeads, usually magnetic, that are coated with an antibody that recognizes certain markers present on the EV surface. This technology can be used for EV isolation from either cell culture media or body fluids. After mixing the EV sample with the antibody-coated microbeads, a magnetic force is applied (i.e., to a column, microplate) which retains the EV-covered microbeads, while the rest of the sample is discarded (www.systembio.com/exosomes). Next, the microbeads with attached EV are eluted using appropriate buffers and used for further analysis. The advantage of this isolation method is its ability to select a specific EV population based on a marker expression regardless of its size. Many groups put in a lot of effort into selecting an EV fraction (i.e., exosomes and microvesicles) based on EV size, but not on the surface marker profile. Although this approach may result in obtaining valuable information on such EV fractions, it limits, however, the overall scope on the impact that the whole EV population, regardless of size, may exert on the surroundings. This is particularly evident in an *in vivo* setting where different types of cells may generate EVs of different sizes, but still carry the same surface marker (10). Another important advantage is the ability of coupling this method with other methods (i.e., flow cytometry, western blotting and ‘real-time’ PCR) to characterize even further the already selected/specific EV fraction. At the same time, however, it needs to be pointed out that the beads, due to their physical binding surface area, can only bind a certain number of EVs, which, in turn, can lead to a substantial population of EVs being still left out from the sample or lost during the purification process.

An alternative to magnetic microbeads are surfactant-free latex beads typically made of polystyrene (49), which are stabilized against aggregation by covalently linked charge groups (sulfate, carboxyl, amidine, carboxyl/sulfate, aldehyde/sulfate, chloromethyl and aldehyde/amidine) and have ~95% of their surface available for passive adsorption of proteins (www.Invitrogen.com). They can be either hydrophobic or hydrophilic and can covalently bind EVs regardless of their size or surface marker composition. Due to this feature, the bound EVs can be further characterized by, for example, flow cytometry using multi-antibody staining, thus providing valuable information on the EVs. Although this method can lead to the acquisition of informative data on EVs, their elution from the beads is difficult. This is a major disadvantage to this method, since the bound EVs cannot be used in downstream experiments.

### Size exclusion chromatography

This method is usually coupled with a low-speed centrifugation step that allows the removal of larger objects from the sample (cells, cellular debris, organelles, etc.) that is followed by a filtration step (0.8 and 0.2 *µ*m pore size filter) to pre-concentrate the EVs. The filtered EV sample is then subjected to size exclusion chromatography (usually gel filtration column) where small volume fractions (1 ml) of the filtrate are collected and ultracentrifuged (100,000 × g, 1 h and longer) to pellet down the EVs (50–52). The principal behind this technique is that particles in a sample, depending on their size, will move through the filtration column at different rates. Thus, larger particles will elute more rapidly, while the smaller ones more slowly, due to their ability to penetrate the stationary phase (gel) of the column. In theory, the obtained eluted fraction at a certain time should contain a population of particles of the same particle size. The pelleted EVs are resuspended in PBS and used in downstream assays.

Although this method is generally established as one of the methods for EV isolation, it poses some concerns that need to be addressed. Forcing EV passage through filters used to pre-concentrate the sample may lead to EV deformation and eventual rapture into smaller particles (30). To avoid this, it has been suggested that size exclusion chromatography should be performed by gravity or with the application of the smallest possible force (23,30). Moreover, the selection of the appropriate gel type is crucial to the recovery of EVs, rather than proteins or lipoproteins. Additionally, the short isolation time and relatively low cost are also beneficial.

## 7. Polymeric precipitation

Although polymeric precipitation methods do not fall under the isolation method classification proposed above, it is worthwhile to mention them as an interesting and promising alternative. The concept behind this method is the formation of a meshlike polymeric web that captures EVs of a certain size, usually between 60–180 nm, which are later pelleted at low centrifugal speeds. It can be used to obtain EVs from both cell culture medium and/or body fluid samples. What is very appealing about this EV isolation method is the fact that it is relatively quick, enables high EV recoveries and does not require laborious ultracentrifugation (53). A previous study showed the superiority of this method over others; however, it only assessed the RNA yield and protein purity/quantity (54); thus, its possible application in the overall EV recovery still needs to be addressed. Others have raised a concern regarding the contaminants, such as lipoproteins, that may be isolated along with the actual EVs (30). Moreover, since polymeric precipitation isolates EVs of 60–180 nm in size, it cannot be used for the assessment of larger EVs present in a sample.

## 8. Biological activities of EVs and associated challenges

It is rather difficult to evaluate how the different isolation protocols affect the potential biological activities of EVs. The main reason for this is that the majority of available data concerns EVs isolated by a single method, and this mostly involves differential centrifugation/ultracentrifugation. At the same time, studies focusing on the comparison of the biological effects of EVs isolated by more than one protocol simply are unavailable, at least to the best of our knowledge. Most groups choose one isolation protocol through which EVs are obtained and their effect is then evaluated depending on the experimental design. For instance, exosomes isolated from red blood cells by ultracentrifugation are able to induce the release of pro-inflammatory cytokine in monocytes (55). Another study demonstrated that density-gradient isolated glioblastoma-derived EVs modified the phenotype of monocytic cells (56). It has also been shown that RNA transferred by macrophage-derived microvesicles isolated by ultracentrifugation is biologically active and induces macrophage differentiation (57). Although there are attempts to compare two or more EV isolation methods, the results are limited to the correlation of EV physical properties (e.g., number, size distribution, phenotype, protein and/or RNA expression), and do not show the biological effects. For example, in a study on EVs present in blood and urine, it was suggested that polymeric precipitation yielded the highest EV concentration, although CD133 and CD63 protein expression in these EVs was difficult to interpret compared to EVs obtained by other methods (58). What makes the assessment of EV biological activities even more difficult is that there is no single isolation protocol to refer to as the ‘golden standard’ guaranteeing not just the complete recovery of EVs (all the fractions), but also the recovery of EVs that retain their native form/shape and function. Thus, it is simply unknown as to whether the obtained EVs, using either one of the protocols discussed above, comprise the original EVs released by the parent cells. Due to these discrepancies, additional simultaneous studies on the effects of different isolation methods are warranted for the proper evaluation of EV biological activities.

## 9. Conclusion

The past decade has witnessed an extensive research in the EV field. As we learn more about EVs and their role under normal and/or pathological conditions, there is an increasing temptation of trying to use that knowledge in the treatment of certain diseases. Although elevated numbers of EVs or EVs bearing certain proteins, RNAs, miRNAs, etc. have been tagged as diagnostic and prognostic markers in a number of diseases, there still seems to be a question pending as to the validity of this information. The answer to this question begins with the establishment of firm EV isolation protocol(s) that would enable the assessment of the full spectrum of EVs present in a sample instantaneously. Available isolation protocols only in part meet this goal, as they tend to focus on the selection of a certain type(s) of EV, whether based on their size, density or marker expression. The outcomes of these approaches produce either inadequate EV numbers/concentration or contaminated EV population(s)/fraction(s), whose usefulness in downstream tests may be questionable. The isolation methods presented in this review comprise the current knowledge on the topic, shedding a light on the complexity of the EV ‘world’.
